# Primary perianal Paget’s disease: three cases

**DOI:** 10.1093/jscr/rjad684

**Published:** 2023-12-28

**Authors:** Erika Lydrup, Marie-Louise Lydrup, Erik Agger

**Affiliations:** Department of Dermatology and Venerology, Skåne University Hospital (SUS Malmö), Jan Waldenströms gata 18, S-205 02 Malmö, Sweden; Department of Surgery, Colorectal Unit, Skåne University Hospital (SUS Malmö), S-205 02 Malmö, Sweden; Department of Surgery, Colorectal Unit, Skåne University Hospital (SUS Malmö), S-205 02 Malmö, Sweden

**Keywords:** perianal Paget’s disease, Paget’s disease, extramammary Paget’s disease

## Abstract

Perianal Paget’s disease (PPD) is a rare intraepidermal neoplastic disease, presenting with nonspecific symptoms, such as pruritis ani or eczema. Perianal Paget’s disease may present as a primary lesion or as a paramalignant phenomenon. Uniform evidence-based treatment strategies have not been defined for this rare condition, and currently, different treatment methods are suggested. This case report presents three cases of perianal Paget’s disease with three different treatments and outcomes. Pathogenesis, treatment, and the importance of a strict follow-up program are discussed.

## Introduction

Perianal Paget’s disease (PPD), a form of extramammary Paget’s disease (EMPD), is an uncommon intraepidermal neoplastic disease. PPD can be a paramalignant phenomenon or, more uncommonly, a primary lesion without underlying malignancy [1, 2]. The symptoms are usually nonspecific (e.g. pruritis ani or an eczematous lesion), often leading to misdiagnosis and delayed diagnosis. The condition and its treatment have not been studied in comparative studies, and current evidence does not support any one uniform treatment strategy.

In this paper, three cases of PPD with different treatments are presented: surgical excision combined with topical treatment, radiotherapy (RT), and one case of palliation due to metastatic disease. The patients presented in this series were all investigated and found negative for other primary malignancies.

### Case 1

**Figure 1:**
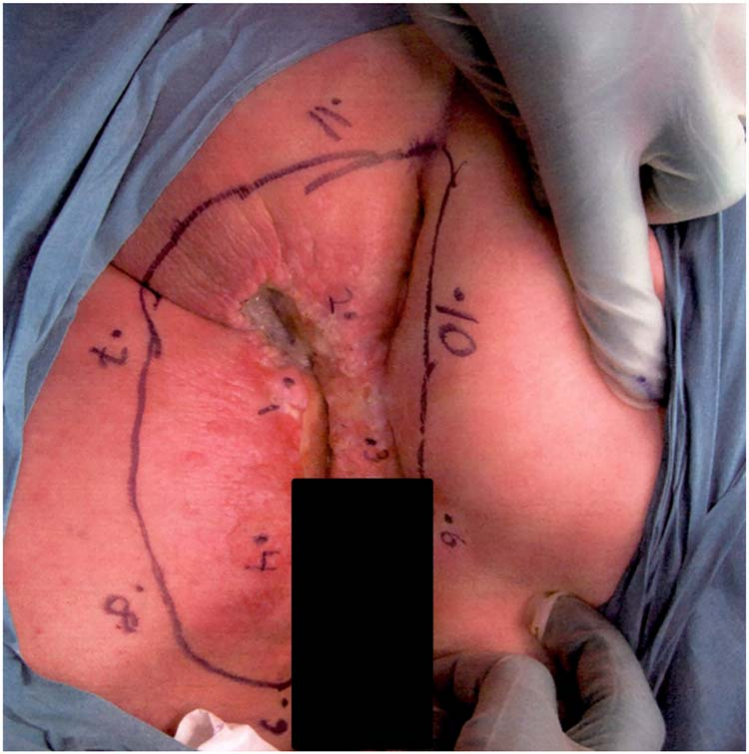
Photograph of the affected lesion in our first patient. Mapping biopsies confirms Paget’s disease in macroscopically normal tissue.

An 83-year-old man with a history of hypertension and asthma but no previous abdominal surgery was referred to the Department of General Surgery with an 8 × 8 cm perianal necrosis at the scrotal base (Fig. 1). Nineteen years earlier, the patient had been diagnosed with PPD without invasiveness located in the same area and treated with RT twice over 4 years with a total radiation dose of 82 Gy. The patient was examined by colorectal surgeons, plastic surgeons, and urologists. The lesion was located at the scrotal base, with a necrotic ulcer in the perianal area. Initially, the condition was interpreted as a side effect of RT, but, after multiple biopsies, histopathological examination showed Paget without signs of invasiveness.

Clinical workup including normal colonoscopy and MRI of the lower abdomen excluded involvement of the urethra or corpora cavernosum. FDG-PET-CT demonstrated hypermetabolism in the perianal area and in both groins, but no findings indicated malignancy elsewhere. The multidisciplinary conference recommended an abdominoperineal rectal excision, including a muscle flap for perineal closure. Before surgery, multiple mapping biopsies of surrounding macroscopically normal tissue also showed Paget’s disease.

The surgical procedure included resection of the damaged skin in the perineum, scrotum, and left thigh in addition to the abdominoperineal resection. The defect was closed with bilateral gluteal fasciocutaneous flaps. Postoperatively, the patient developed phlegmon and flap separation and was successfully treated with vacuum-assisted closure and antibiotics. The histopathological examination showed Paget in squamous epithelium with no invasiveness but not radically removed as expected. Seventeen months after surgery, a biopsy from the pubic area was taken, again showing Paget’s disease, and Aldara^®^ treatment with an advantageous outcome was started. The patient later relapsed with EMPD in the same area, needing repeated symptomatic treatment with a CO_2_ laser with good results. Today, the patient is 89 years old, in good health, and still participating in a control program at the Department of Dermatology.

### Case 2

**Figure 2:**
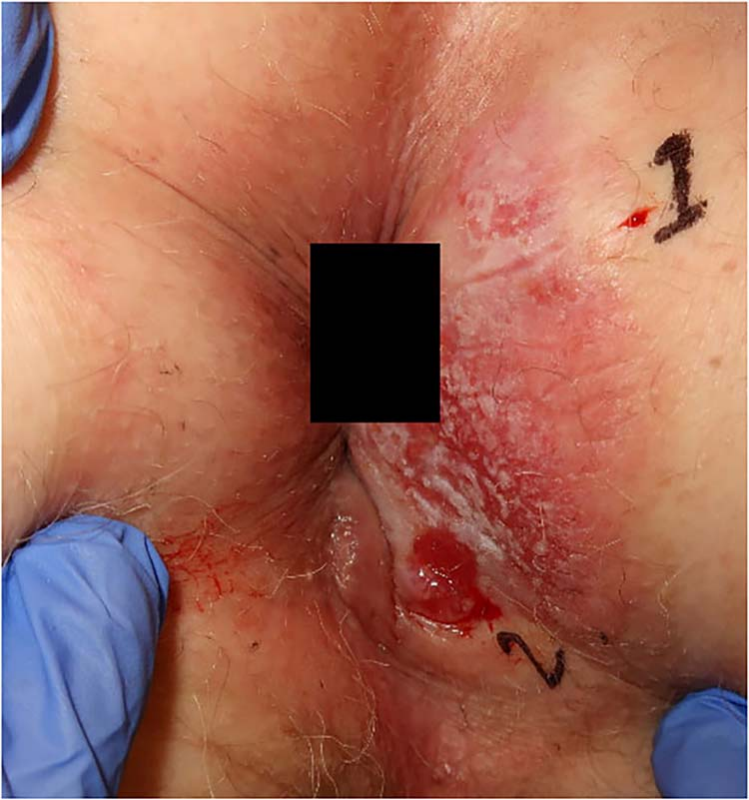
Photograph of the lesion in our second patient. Biopsies confirming Paget’s disease in biopsy marked 1 and Paget with underlying anal gland carcinoma in biopsy marked 2.

An 84-year-old man with a history of hypertension, hyperlipidemia, sleep apnea, and an earlier transient ischemic attack was referred with a history of itching and bleeding for 6 months from a perianal rash. The lesion was 5 × 6 cm, with a central ulceration of 2 × 2 cm (Fig. 2). Histopathological examination showed Paget in one biopsy and Paget with underlying anal gland carcinoma in the other. At a multidisciplinary conference, the lesion was interpreted as adenocarcinoma from the cryptal glands of the anal canal, secondary to the Paget.

Rigid sigmoidoscopy visualized a 3 mm adenoma and hemorrhoids, but was otherwise normal. CT thorax-abdomen showed bladder diverticula but no metastases. An MRI of the rectum presented a superficial tumor and a 13-mm pathological lymph node in the right groin. PET-CT confirmed pathological metabolism in the right groin but no other signs of metastases. The patient was treated with long-course RT (45 Gy/25 fractions) combined with capecitabine. At reevaluation 1 month later, no visible tumor could be seen. MRI of the rectum showed regress and no suspect glands in the groin, and FDG-PET-CT showed complete metabolic regression in the perianal area and in the right groin. At the revisit 3 months later, no visible tumor could be seen, and FDG-PET-CT was performed without signs of relapse. Unfortunately, the patients developed liver and skeletal metastases from the anal gland carcinoma after 21 months and died 3 months later.

### Case 3

**Figure 3:**
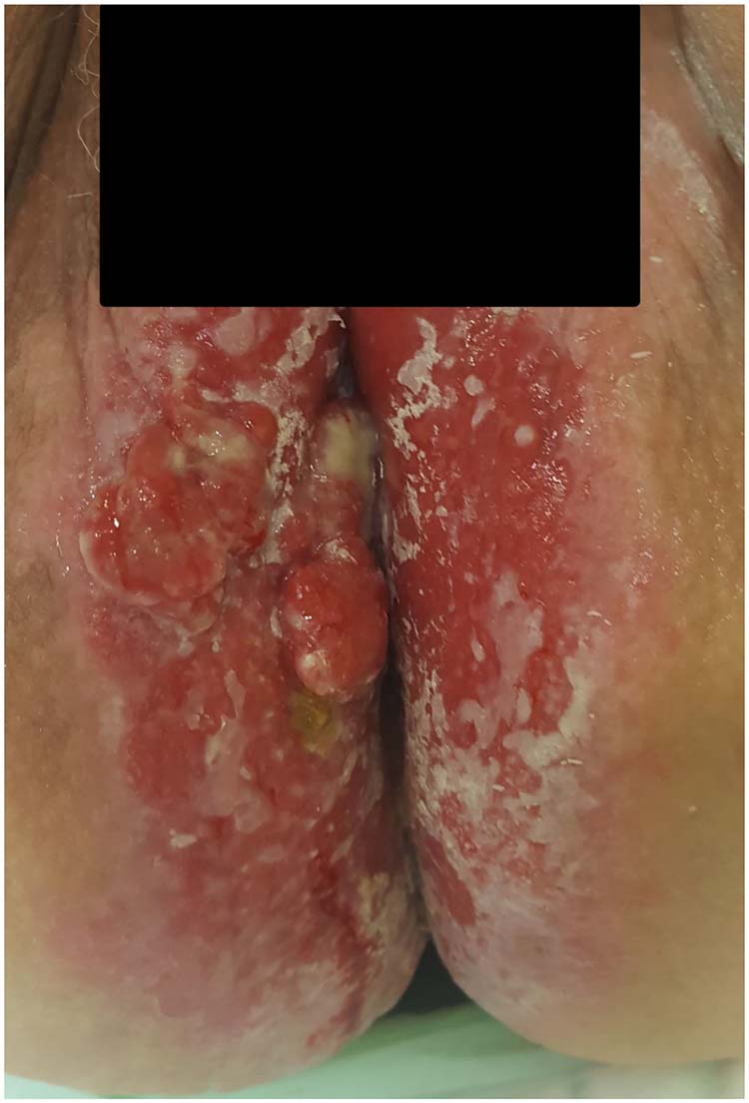
Photograph of the area with severe perianal dermatitis in our third patient. Biopsies show primary EMPD with invasion.

A 66-year-old woman with kidney failure and peritoneal dialysis, diabetes, hypertension, stool incontinence, severe physical disability, and cognitive dysfunction was referred to the Department of Gynecology with severe dermatitis in the perineum and suspected anal cancer. Examination showed aggressive dermatitis 1–1.5 cm in diameter in the perineum, 1.5 cm from the anal opening (Fig. 3). Biopsies showed primary EMPD with invasion. The colonoscopy was incomplete. And CT thorax and abdomen are normal. At revisit 1 month later, the dermatitis had grown to a size of 2 cm in diameter. A multidisciplinary conference recommended radical local excision. However, the lesion progressed rapidly, and the patient was recommended for abdominoperineal rectal excision, but declined this treatment. Palliative excision was planned, although several new lesions were discovered and biopsies showed Paget’s disease in all biopsies, both with and without invasiveness. Despite disease progression, the patient declined further treatment and died 5 months later due to peritoneal-dialysis-related peritonitis.

## Discussion

### Background

PPD is an uncommon intraepidermal neoplastic disease mostly seen in patients between 60 and 70 years of age. The condition is often associated with an underlying malignancy in the rectum, stomach, breast, or ureter, which has to be excluded [1, 2].

PPD most often arises as a primary cutaneous neoplasm (primary PPD) and is less commonly spread from an underlying gastrointestinal or genitourinary cancer (secondary PPD) [1, 2]. If left untreated, primary PPD may progress to locally invasive carcinoma or even metastasize to lymph nodes or distant sites [3, 4].

### Symptoms

The clinical presentation of PPD is usually nonspecific with perianal pruritis, pain at defection, and an eczematous, pink or reddish plaque with well-defined borders. The condition is often misdiagnosed, and the patient can have symptoms for years before treatment is initiated [2, 4].

Differential diagnoses include seborrheic dermatitis, superficial fungal infections, Bowen’s disease, melanoma, basal and squamous cell carcinoma, dermatitis, eczema, and psoriasis [2–4].

If a chronic dermatosis does not respond to standard topical therapy, a biopsy should always be considered to rule out PPD and other neoplasms [4].

### Histopathology

The diagnosis of PPD is defined by a full-thickness skin biopsy [4]. Microscopically, the lesion is characterized by large cells with pale-staining, basophilic cytoplasm, and large round hyperchromatic nuclei, called Paget’s cells*.* To identify Paget’s cells and differentiate between the primary and secondary forms of PPD, immunohistochemical staining is used [5].

### Treatment

The treatment of PPD depends on the extent and depth of the invasion and whether lymph node involvement or systematic spread is present [1]. If the patient is fit for a surgical procedure, surgical resection as primary therapy is recommended [4, 6].

Nonsurgical treatments may be offered to patients unfit for surgery or preoperatively to downsize the area with confirmed PPD and as adjunctive therapy to surgery in patients with advanced disease [7].

### Surgical treatment

Historically, the standard surgical treatment for primary PPD has been wide local excision (WLE) with at least a 1 cm margin [1]. To determine margin status, intraoperative pathological examination of frozen sections is recommended [7]. Unfortunately, due to the multifocal nature of PPD with irregular shapes and indistinct borders, resection margins are often positive in later permanent histological analyses [1, 4]. Due to this, Mohs micrographic surgery, a technique, where the surgeons microscopically examine the entire margins of the excision, is more often recommended, especially in areas with functionally important anatomic structures [4, 6]. The procedure is, however, time-consuming, and distinguishing Paget cells microscopically can be challenging [7].

For secondary PPD, more extensive surgery with wide local excision of the cutaneous affected area is recommended, and in some high-risk cases with inguinal lymph node dissection [6].

When primary closure is not possible, other options are skin grafting, flap transpositions, or leaving the wound open for secondary healing with or without the use of a vacuum device [1]. Complications, like anal stricture, anal ectropion, and graft failure, are common [8].

### Nonsurgical treatment

Surgical excision of PPD may result in the removal of large areas of skin, and alternative nonsurgical treatment strategies have been explored [4, 7].

### Photodynamic therapy (PDT)

PDT is a successful treatment for superficial epidermal neoplasms [7].

PDT treatment consists of methyl-5-aminolevulinate or 5-aminolevilunic applied topically or porfimer sodium administered intravenously. Visible light is applied, activating the photosensitizing agent and causing selective destruction of neoplastic tissue [5].

PDT may be beneficial when treating large areas and in patients who are not suitable for surgery. The advantages of PDT include cancer selectivity, with the potential to treat skip areas, and a good cosmetic outcome [4]. It may be used either as primary therapy or in combination with surgery [7].

### Topical treatment

Imiquimod is a topical immunomodulatory cream that stimulates cytokines at the site of application, resulting in a specific immune response to tumor cells [4, 5, 7].

5-FU is at a pyrimidine analog that inhibits the synthesis of DNA and RNA*.* It has been shown to be effective in downsizing lesions prior to surgery but does not appear to be effective as a single therapy for PPD [4, 5].

### Radiotherapy

RT can be used as primary treatment in patients unsuitable for surgery but also in cases of recurrence, large burden of disease or in combination with surgery. A total dose of at least 50 Gy is often used [1]. RT may be beneficial in selected patients, as in our second case, but the risk of toxicity and impaired wound healing, as in our first case, should be considered.

### Chemotherapy

Chemotherapy is a possible treatment method for patients with metastatic disease but is not frequently used, and the effect has not been systematically evaluated [7]. Some reports have shown a successful outcome after treatment with combination chemotherapy consisting of mitomycin C, epirubicin, vincristine, cisplatin, and 5-fluorouraci [9, 10].

### Prognosis

The prognosis of PPD depends on the extent of the disease, and it is well known that patients with in situ disease have a more favorable prognosis than patients with invasive disease [4]. Karam *et al.* [11] investigated the outcome of patients diagnosed with EMPD between 1973 and 2000. In all, 86.4% of the patients were treated with directed surgery, 6.4% with RT, and 9.7% of the patients did not undergo either surgery or radiation therapy. The 5-year disease-specific survival (DSS) was 94.9% for patients with localized disease, compared to 84.9% for patients with regional disease and 52.5% for patients with distant disease. Shorter DSS was associated with older age and advanced-stage treatment modality.

### Follow-up

Recurrence rates of PPD are high, probably due to the clinical features of PPD, including irregular margins and the tendency to involve apparently normal skin, making radical resection challenging [12]. Long-term follow-up is necessary.

For patients diagnosed with primary noninvasive EMPD, Lam *et al*. [5] recommend clinical evaluation twice a year for the first 3 years, then once a year. Internal malignancy screening should be directed toward particular signs or symptoms. In secondary EMPD, a closer follow-up three to four times a year is motivated, with screening directed to the site of the lesion once a year [5].

## Conclusion

PPD is a rare and often misdiagnosed disease in elderly patients presenting with perianal itching and eczema. It may be associated with gastrointestinal or genitourinary malignancies, which must be excluded. The standard recommendation for treating PPD is WLE with at least 1 cm margin, but nonsurgical methods, such as imiquimod cream, PDT, and RT might be used. Regardless of the therapeutic method chosen, long-term follow-up is necessary due to the high risk of recurrence.

In this paper, we present three cases of PPD. The first case demonstrates that RT as a treatment option is not always without risks and that surgery in combination with TT may be a feasible option. Our second case presents the opposite, namely complete regression of PPD after RT and no side effects of the treatment. This treatment made it possible for the patient to avoid surgery and a stoma. Our third case demonstrates how aggressive PPD can be and that meticulous examination and early diagnosis are crucial for disease control.
